# Frequency of occurrence of polymorphic light eruption in patients treated with photohardening and patients treated with phototherapy for other diseases

**DOI:** 10.1111/phpp.12429

**Published:** 2018-11-13

**Authors:** Alexandra Gruber‐Wackernagel, Angelika Hofer, Franz Legat, Peter Wolf

**Affiliations:** ^1^ Research Unit for Photodermatology Department of Dermatology Medical University of Graz Graz Austria

**Keywords:** photohardening, phototherapy, polymorphic light eruption, psoriasis

## Abstract

**Background:**

Medical phototherapy can lead to the manifestation of polymorphic light eruption (PLE), though little is known about the frequency of such events.

**Aims:**

The aim of this Austrian single center study was to retrospectively investigate over a 4‐year time period the frequency of PLE in patients prone to the condition and patients with other diseases under phototherapy (mainly narrow‐band and broad‐band UVB).

**Materials and Methods:**

The data for analysis were obtained from the electronic health and patient record database and patient files of the Photodermatology Unit, Department of Dermatology, Medical University of Graz, Austria.

**Results:**

PLE occurred in 24.3% (18/74) of PLE patients but only 0.7% (3/421) of psoriasis patients under phototherapy (chi‐square; *P* < 0.0001). PLE also occurred in 1.2% (3/257) of patients with atopic eczema, 0.8% (1/118) with prurigo, 3.5% (4/115, *P* = 0.0206) with parapsoriasis en plaques/mycosis fungoides, 7.4% (2/27, *P* = 0.0013) with granuloma anulare, 14.3% (1/7, *P* = 0.0002) with scleroderma, and 16.7% (1/6, *P* < 0.0001 vs. psoriasis) with pityriasis lichenoides chronica or pityriasis lichenoides eruptiva et varioliformis acuta.

**Discussion and Conclusion:**

These results are helpful for treatment allocation and risk estimation of PLE occurrence with regard to obtaining informed consent not only from PLE‐prone patients but also from patients with other skin disorders commonly treated by phototherapy.

## 1 INTRODUCTION

Polymorphic light eruption (PLE) is the most common photodermatosis and is especially prevalent among young women in temperate climates.^1^ Severely affected individuals, who experience repeated attacks of PLE throughout the summer, may require prophylactic medical photohardening each spring before the first intense sun exposure. Medical photohardening simulates the naturally occurring phenomenon of hardening and aims to induce photoadaption. Broadband UVB (290‐320 nm) (BB‐UVB), narrowband UVB (311‐313 nm) (NB‐UVB), and psoralen plus UVA (PUVA) photochemotherapy are effective in photohardening of PLE.^1^, ^2^, ^3^, ^4^, ^5^, ^6^, ^7^ Although it is well known that medical phototherapy can lead to PLE, little is known about the frequency of such events. The aim of this study was to investigate the frequency of PLE under prophylactic photohardening in PLE‐prone patients. Additionally, we investigated the frequency of PLE during phototherapy in patients with other diseases, including psoriasis, atopic eczema, prurigo, parapsoriasis en plaques and mycosis fungoides, pityriasis lichenoides chronica (PLC) or pityriasis lichenoides eruptiva et varioliformis acuta (PLEVA), granuloma anulare, and scleroderma.

## 2 MATERIALS AND METHODS

### 2.1 Study design

This was a retrospective case study of the occurrence of PLE over a 4‐year period (from January 2013 to December 2016) in patients treated with phototherapy at the Photodermatology Unit, Department of Dermatology, Medical University of Graz, Austria. The data for analysis were obtained from the electronic health and patient record database and patient files of the Photodermatology Unit. The study was approved by the Ethics Committee of the Medical University of Graz through protocol number 25‐294 ex 12/13.

### 2.2 Patient identification

First, we electronically searched the database to identify patients (irrespective of primary diagnosis) who underwent phototherapy and exhibited PLE under treatment. Key search terms were “polymorphous,” “polymorphic,” “PLE,” and “PLD” (ie, the German abbreviation of PLE) in the data fields of “diagnosis,” “patient history,” and “course of disease” in the database. The patient records so identified were then screened for prior history from 1998 onward. Second, we electronically searched the database for records of all patients with the specific diagnoses listed in Table 1 in whom PLE had occurred and who had been identified in the first step of our electronic database search. The key words for the diagnoses were the primary designation of a specific diagnosis (eg, psoriasis); in addition, for each diagnosis, we used related abbreviations and characteristic, truncated parts of diagnostic terms (such as “psor” and others) to identify and include the data of patients whose diagnoses may have been potentially misspelled in the database. Then, we performed automatic and manual data extractions to obtain patient demographics, history, and phototherapy characteristics.

**Table 1:** Frequency of PLE manifestation during phototherapy in patients with different diseases

Primary diagnosis	Number of patients (females/males) with PLE at any phototherapy cycle/total number of patients (females/males)^a^	Number of phototherapy cycles with occurrence of PLE/total number of phototherapy cycles				
		All wavebands	NB‐UVB	BB‐UVB^b^	PUVA	UVA1
PLE	18 (16/2)/74 (65/9) (24.3) *P* < 0.0001	26/130 (20.0)	22/113 (19.5)	4/13 (30.8), *P* = 0.34	0/4 (0)	–
Other diagnoses						
Atopic Eczema	3 (1/2)/257 (123/134) (1.2) *P* = 0.54	3/339 (0.9)	1/212 (0.5)	0/8 (0)	1/36 (2.8)	1/83 (1.2)
Erythroderma	1 (0/1)/23 (9/14) (4.3) *P* = 0.07	1/29 (3.4)	1/7 (14.3)	–	0/20 (0)	0/2 (0)
Granuloma anulare	2 (1/1)/27 (21/6) (7.4) *P* = 0.001	3/35 (8.6)	0/2 (0)	–	2/24 (8.3)	1/9 (11.1)
Parapsoriasis en plaques, Mycosis fungoides	4 (2/2)/115 (24/91) (3.5) *P* = 0.02	4/180 (2.2)	2/82 (2.4)	–	2/98 (2.0)	–
PLEVA/PLC	1 (0/1)/6 (2/4) (16.7) *P* < 0.0001	1/6 (16.7)	0/2 (0)	–	1/4 (25.0)	–
Prurigo	1 (0/1)/118 (73/45) (0.8) *P* = 0.88	1/162 (0.6)	0/117 (0)	0/18 (0)	1/12 (8.3)	0/15 (0)
Psoriasis	3 (2/1)/421 (181/240) (0.7) n.a.	3/686 (0.4)	3/552 (0.5)	0/1 (0)	0/133 (0)	–
Scleroderma	1 (1/0)/7 (5/2) (14.3) *P* = 0.0002	1/11 (9.1)	–	–	0/1 (0)	1/10 (10.0)
Total of other diagnoses	16 (7/9)/974 (438/536)	17/1448 (1.2%)	7/974 (0.7%) –	0/27 (0.0%), *P* = 0.658	7/328 (2.1%) –	3/119 (2.5%) –

Frequency given as percentage in parentheses.

a *P* value was determined by Fisher exact or chi‐square test, comparing the frequency of PLE manifestation in a certain disease vs that in psoriasis.

b *P* value was determined by Fisher exact test, comparing the frequency of PLE manifestation in NB‐UVB vs BB‐UVB cycles. –: none; n.a.: not available.

### 2.3 Statistical analysis

Descriptive data from our analyses were presented in tables. The Fisher exact or chi‐square test, as appropriate, was used to compare the prevalence of PLE under phototherapy between different groups of patients. A *P* value *P* < 0.05 was considered significant.

## 3 RESULTS

Our electronic database search identified the occurrence of PLE under photohardening (in any cycle) in 24.3% (18/74) of PLE patients (Table 1). Photohardening with suberythemal dosages of NB‐UVB had been administered to all PLE patients during phototherapy treatment cycles 2‐3 times per week for 4‐6 weeks in spring, at a starting dose of 0.2 J/cm^2^ and in dose increments of 0.05–0.1 J/cm^2^ per treatment, as tolerated.^8^ The other forms of phototherapy had been administered to the PLE patients, as previously described.^7^ The frequency of PLE in patients with other diseases treated with minimal photoxic dose‐ or skin phototype‐based suberythemal phototherapy ranged from 0.4% (psoriasis) to 16.7% (PLEVA). PLE occurred significantly more often in PLE‐prone patients who underwent photohardening than in psoriasis patients who underwent phototherapy (*P* < 0.0001). Compared with its occurrence in patients with psoriasis (the disease with the lowest observed prevalence of PLE), PLE occurred significantly more often under phototherapy in patients with parapsoriasis en plaques/mycosis fungoides (3.5%, *P* = 0.0206), granuloma anulare (7.4%, *P* = 0.0013), scleroderma (14.3%, *P* = 0.0002), and PLEVA (16.7%, *P* < 0.0001) (Table 1). The frequency of PLE in patients with atopic eczema and prurigo was low and did not differ statistically from that in patients with psoriasis. PLE occurrence did not appear to be affected by wavebands or phototherapeutic modalities administered to the patients (Table 1). The demographics and phototherapy characteristics of PLE patients undergoing phototherapy are presented for individual patients in Table 2 and then summarized in Table 3. Under daily life conditions and exposure to natural sunlight, more women than men experienced PLE under photohardening (ie, 16 vs 2) (Table 1), consistent with the observation that more women than men overall had undergone photohardening. Surprisingly, however, the female/male ratio in non‐PLE patients (7/9) was more balanced, despite a similar overall number of phototherapy‐treated women and men in non‐PLE patient groups. Besides, there were no major differences in phototherapy characteristics between PLE patients and non‐PLE patients. For instance, in PLE patients, the disease manifested after a median of five treatment exposures in the first cycle of occurrence, compared with 5.5 treatment exposures in non‐PLE patients (Table 3). In general, the occurrence of PLE in a patient did not result in a change of the allocated standard treatment protocol, though some of them received short‐term topical steroids to mitigate the rash.

**Table 2:** Demographics and phototherapy characteristics of individual PLE patients undergoing photohardening

PLE patient no.	Gender	Skin phototype	Morphology of PLE lesions	Predilection body site	Disease duration at PLE onset during phototherapy, years	PLE occurrence at cycle number (exposure number)/total number of cycles per waveband
1	f	3	macpap	VN, N, FA,T	13	1 (1)/1 NB‐UBV
2	f	3	mac	VN, T, UA, FA	29	1 (15), 7 (3)/7 NB‐UVB
3	f	3	urt	VN, UA, N, AB	6	3 (10), 4 (7), 6 (2,7)/6 NB‐UVB
4	f	1	urt	VN, FA	16	1 (1)/1 NB‐UVB
5	f	2	urt	VN, N, UA, FA	5	2 (5)/3 NB‐UVB
6	f	3	macpap	FAC, N, VN, UA, FA, L, H,F	40	1 (1)/1 BB‐UVB, 0 (0)/3 NB‐UVB
7	m	3	pap	VN	3	1 (1,4,16), 2 (7,9)/2 NB‐UVB
8	f	3	pap	FAC, N, VN, AB, UA, FA, T	5	1 (3)/1 NB‐UVB
9	f	3	papves	VN, UA, FA, T, L	14	1 (2,6)/1 NB‐UVB
10	m	3	pap	FA, VN, B, T	2	1 (11)/1 BB‐UVB, 1 (5)/1 NB‐UVB
11	f	na	pap	VN, FA, UA	19	1 (5,10,14)/1 BB‐UVB
12	f	na	papves	VN, UA, FA, L, FAC, B	8	1 (6)/1 NB‐UVB
13	f	2	macpap	FA, VN, T	20	1 (10), 2 (12), 4 (8)/4 NB‐UVB
14	f	3	papves	VN, UA, FA	16	1 (14)/1 NB‐UVB
15	f	3	pap	VN	6	1 (9), 2 (9)/2 NB‐UVB, 0 (0)/3 PUVA
16	f	na	pap	FAC, VN, UA, FA, AB	9	1 (12)/1 BB‐UVB
17	f	na	pap	VN	19	1 (2)/1 NB‐UVB
18	f	3	mac	VN, T	8	1 (2,11)/1 NB‐UVB

VN: V‐neck; N: neck; FA: forearm; UA: upper arm; T: thigh; AB: abdomen; FAC: face; L: lower leg; H: dorsal hands; F: dorsal feet; B: back; mac: macular; pap: papular; ves: vesicular; urt: urticarial.

**Table 3:** Summary of demographics and treatment characteristics of patients with PLE eruption occurring under phototherapy

Primary diagnosis	Patient number	Gender	Median age (range), years	Skin phototype	Morphology of PLE lesions	Predilection body site	Median (range of) disease duration at PLE onset during phototherapy, years	Number of patients with one or more positive cycles	First PLE eruption at phototherapy cycle number	Number of PLE positive cycles per total number of cycles	Median exposure number (range of) with PLE occurrence in first cycle with PLE eruption
Non‐PLE	16	f:7 m:9	54.0 19‐88	II: 1 na: 15	pap:9 macpap:6 papves:1	VN: 10 B: 6 AB: 2 GLUT: 3 T: 3 UA: 2 L: 1 na: 1	n.a.	One positive cycle, *n* = 16	First cycle: 13/16 patients, second cycle: 3/6 patients*	*n* = 16/27, NB‐UVB: 7/11 BB‐UVB: 1/2 PUVA: 5/11 UVA1: 3/3	5.5 (1‐34)
PLE	18	f:16 m:2	41.3 19‐64	I: 1 II:2 III:11 na:4	mac:2 macpap:3 urt:3 papves:3 pap:7	VN: 18 N: 5 FA:13 T: 7 UA: 10 AB: 3 FAC: 4 L: 3 H: 1 F: 1 B: 2	10.7, (2‐40)	One positive cycle, *n* = 12, two positive cycles, *n* = 4 three positive cycles, *n* = 2	First cycle: 16/18 patients, second cycle: 1/8 patients, third cycle: 1/6 patients^a^	*n* = 26/43, NB‐UVB: 22/36 BB‐UVB: 4/4 PUVA: 0/3	5.0 (1‐15)

VN, V‐neck; N, neck; FA, forearm; UA, upper arm; T, thigh; AB, abdomen; FAC, face; L, lower leg; H, dorsal hands; F, dorsal. feet; B, back, GLUT, gluteal mac, macular; pap, papular; ves, vesicular; urt, urticarial; *6 of the 16 patients had two or more (up to five) phototherapy cycles.

a Three of the 18 patients had two or more (up to three) phototherapy cycles.

## 4 DISCUSSION

In this Austrian study, PLE occurred in 24% (18/74) of PLE patients under photohardening, a percentage considerably lower than the 56% (44/79) recently reported in a 5‐year single‐center, case‐series review from Scotland.^5^ The reasons for this difference remain elusive, but may be partly due to differences in phototherapy treatment protocols and/or skin phototype. In our study, the minority (21%, 3/14) of phototyped PLE patients who exhibited disease symptoms under photohardening were of skin phototype I/II, compared with the majority (76%, 60/79) in the Scottish study.

Notably, our results showed a very low prevalence of PLE in psoriasis patients undergoing phototherapy (0.7%, [3/421]), even though PLE in psoriasis might not be so rare in the general population.^9^ PLE seems to be highly linked to photosensitive variants of psoriatic disease. In an epidemiologic study, 43% of all patients suffering from photosensitive psoriasis were found to have a history of PLE with secondary exacerbation of psoriatic lesions.^10^ We recently speculated that resistance to UV‐induced immune suppression in PLE may photoaggravate other diseases such as coexisting psoriasis, which commonly responds beneficially to UV radiation from sunlight or artificial sources.^9^ Psoriasis patients who are susceptible to PLE may not experience the beneficial antipsoriatic effects of UVB but may instead experience induction and/or worsening of psoriatic disease after UVB exposure. In these patients, UVB radiation may induce innate immunity through antimicrobial peptides such as cathelicidin LL‐37, resulting in psoriatic lesions when there is simultaneous resistance to the UV‐induced suppression of the adaptive immune response (like in PLE) that would normally counteract such lesions.^9^ Thus, patients with photosensitive psoriasis may not be allocated to UVB phototherapy (based on a history of photoaggravation that is possibly linked to PLE), which may in turn explain the very low prevalence of PLE in our series of psoriasis patients. In contrast, PUVA may act in photosensitive psoriasis without provoking PLE,^11^, ^12^ consistent with our data showing that none of 133 psoriatic patients treated with PUVA developed PLE during treatment (Table 1).

However, we observed a relatively high frequency of PLE under phototherapy in patients with parapsoriasis en plaques/mycosis fungoides (3.5%), granuloma anulare (7.4%), scleroderma (14.3%), and PLC/PLEVA (16.7%). This may be due to differences in pathophysiology and immunological hyperresponsiveness to UV radiation between these conditions and non‐photosensitive psoriasis. We observed PLE in a few non‐PLE patients after UVA1 (340‐400 nm) treatment, including one patient with scleroderma. PLE seems to be induced more often by UVA (320‐400 nm) than by UVB (290‐320 nm), but can also be induced by UVB alone and sometimes by both wavebands together (cited in^2^). The role of UVA in triggering PLE has been well substantiated by phototest findings and the observation that most patients with PLE are sensitive to sunlight through window glass (cited in^2^) and by the observed lack of protection from pure UVB‐absorbing sunscreens.^13^

Although the generalizability of our study's findings is limited by the rather low patient numbers in some of the diseases examined and by the retrospective study design, our findings do suggest that PLE manifestation under photohardening occurs frequently in PLE‐prone patients but variably in other diseases treated with phototherapy. This knowledge will be helpful for allocating treatment, estimating risk of PLE occurrence, and obtaining related informed consent not only from PLE‐prone patients but also from patients with other skin disorders commonly treated with phototherapy.

## CONFLICT OF INTEREST

None declared.
